# The evolution of entomopathogeny in nematodes

**DOI:** 10.1002/ece3.10966

**Published:** 2024-02-13

**Authors:** V. J. Trejo‐Meléndez, J. Ibarra‐Rendón, J. Contreras‐Garduño

**Affiliations:** ^1^ Edificio de Investigación I, ENES, Unidad Morelia, UNAM Morelia Michoacán Mexico; ^2^ Posgrado en Ciencias Biológicas, ENES, Unidad Morelia, UNAM Morelia Michoacán Mexico; ^3^ Centro de Investigación y de Estudios Avanzados del IPN (CINVESTAV) – Irapuato Irapuato Guanajuato Mexico; ^4^ Institute for Evolution and Biodiversity University of Münster Münster Germany

**Keywords:** entomopathogeny, evolution, nematodes, parasitism, Rhabditidae

## Abstract

Understanding how parasites evolved is crucial to understand the host and parasite interaction. The evolution of entomopathogenesis in rhabditid nematodes has traditionally been thought to have occurred twice within the phylum Nematoda: in Steinernematidae and Heterorhabditidae families, which are associated with the entomopathogenic bacteria *Xenorhabdus* and *Photorhabdus*, respectively. However, nematodes from other families that are associated with entomopathogenic bacteria have not been considered to meet the criteria for “entomopathogenic nematodes.” The evolution of parasitism in nematodes suggests that ecological and evolutionary properties shared by families in the order Rhabditida favor the convergent evolution of the entomopathogenic trait in lineages with diverse lifestyles, such as saprotrophs, phoretic, and necromenic nematodes. For this reason, this paper proposes expanding the term “entomopathogenic nematode” considering the diverse modes of this attribute within Rhabditida. Despite studies are required to test the authenticity of the entomopathogenic trait in the reported species, they are valuable links that represent the early stages of specialized lineages to entomopathogenic lifestyle. An ecological and evolutionary exploration of these nematodes has the potential to deepen our comprehension of the evolution of entomopathogenesis as a convergent trait spanning across the Nematoda.

## Graphical abstract

Through convergent evolution, the entomopathogeny in nematodes is widespread within the Rhaditidae families, owing to the shared ecological and evolutionary attributes raised from saprophagy, phoresis, and necromeny.

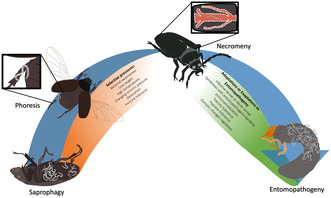

## 1 INTRODUCTION

The phylum Nematoda is recognized for its diversity of species, nearly global distribution, and multifaceted lifestyles (Blaxter & Denver, 2012; Stock, 2015). Among these, parasitism has independently emerged multiple times in the phylum, exhibiting a wide range of traits and strategies (Blaxter & Denver, 2012). For example, some nematodes are giant parasites such as those inhabiting whales, and can reach a length of 8.4 m (Gubanov, 1951). Other nematodes could manipulate the behavior of their hosts, turning them into “zombies” (Davis et al., 2023; Morris et al., 2018). The parasitic nematodes of insects can leap considerable distances to infect them, in some cases using the host's static electricity to propel themselves. The nematodes associated with millipedes feature spines of varying shapes and sizes that enable them to anchor onto their hosts' intestines (Phillips et al., 2020). Even different species of nematodes can collaborate to successfully infect millipedes (Nagae et al., 2021), and the level of cooperation can be even more extreme, as seen with nematodes associated with members of different domains, such as bacteria, to kill their host (Stock, 2015). Parasitic nematodes are ubiquitous within diverse invertebrate and vertebrate species, such as those belonging to the Oxyuridae family, and also affect various plants, including the genera *Heterodera*, *Meloidogyne*, and *Globodera*. Their presence spans both external and internal environments, with some even acting as intracellular parasites, exemplified by *Trichinella spiralis*. These nematodes attract considerable attention due to their pivotal roles in agriculture, veterinary medicine, and human health (Blaxter & Koutsovoulos, 2014; Zuckerman & Rhode, 1981). Conversely, some nematodes are beneficial and used as biological agents for controlling pests in agriculture, forestry, and vectors of human diseases (Gaugler & Kaya, 1990; Grewal et al., 2003). An example are the entomopathogenic nematodes, a particularly valuable and pathogenically interesting group that infect, kill, and feed on insects with the aid of mutualistic bacteria (Dillman et al., 2012; Maher et al., 2017; Navarro et al., 2014; Stock, 2015).

While the term “entomopathogenic” is currently used to describe rhabditids within the families Heterorhabditidae and Steinernematidae, it is evident that other families and orders also have this parasitic attribute such as mermithids (Dorris et al., 1999; Sepulveda‐Cano et al., 2008). The symbiotic relationship between rhabditid nematodes and bacteria is a common occurrence documented in many species. The biology, structure, and mechanisms used by steinernematids and heterorhabditids to kill insects have evolved through convergent evolution (Adams et al., 2006; Murfin et al., 2012). The observed entomopathogenic behavior in species from other families suggests its likely occurrence as a threshold within the Rhabditida order. *Steinernema* and *Heterorhabditis* demonstrate specific and enduring associations, while species such as *Oscheius* or *Rhabditis* exhibit more generalized and transient associations. Given this, entomopathogenicity involves more than two families within the phylum, especially when there is a great number of mermithids parasites of insects. This will help us understand the evolution, scope, and limitations of parasitism within this group and its use for biological control. This paper reviews the evidence suggesting the polyphyletic origin of the entomopathogenic character in Nematoda. First, we will discuss the phylogenetic position and diversity of the phylum. Second, we will explore the general biology of entomopathogenic nematodes (EPN) and posit the ecological evolutionary attributes that promote entomopathogenicity within Rhabditida. Finally, we present a discussion and proposal for the expansion of the definition of “entomopathogenic nematode.” We will show that Nematoda has developed similar biological characteristics with distinct evolutionary histories to kill their hosts.

## 2 PHYLOGENETIC POSITION AND DIVERSITY OF NEMATODA

Nematodes belong to Ecdysozoa, a superphylum characterized by a periodically replaced exoskeleton during their life cycle (Nielsen, 2001). Nematoda is part of the Cycloneuralia group which includes phyla Nematomorpha, Priapulida, Kinorhyncha, and Loricifera (Dunn et al., 2008). Molecular and morphological analyses have revealed that Nematoda is the sister group of Nematomorpha, an exclusive phylum of parasitoids that remain inside the arthropod host during the larval phase and emerge as adults, killing the host in the process (Dunn et al., 2008; Hanelt & Janovym Jr., 1999; Schmidt‐Rhaesa, 1997), similar to mermithids. The proximity between Nematomorpha and Nematoda suggests that nematode ancestors were parasites (Blaxter & Koutsovoulos, 2014). However, molecular data refutes this idea, as free‐living nematodes emerge within the Mermithida order, which is one of the oldest, indicating that the ancestral lifestyle was free‐living and that some lineages subsequently evolved a parasitic lifestyle (Blaxter et al., 1998).

Molecular systematics research on Nematoda has been explored for more than two decades (Blaxter et al., 1998; Kampfer et al., 1998). Based on current molecular analyses, Nematoda is divided into three distinct classes: (A) Enoplia, comprising marine and free‐living nematodes commonly found in marine sediment, which feed on diatoms and seaweeds. This class also includes freshwater and brackish water nematodes, as well as terrestrial and some plant parasitic species; (B) Dorylaimia, encompassing freshwater or terrestrial nematodes that consist of important groups of plant and animal parasites; and (C) Chromadoria, which are marine nematodes, but also feature diverse terrestrial species, plant, and animal parasites. Notably, the order Rhabditida is included in this class (Blaxter & Koutsovoulos, 2014; De Ley & Blaxter, 2002, 2004). Phylogenetic evidence describes multiple independent origins of parasitism in nematodes, appearing at least 18 times among the three classes. Including 3 families of plant parasites, 10 families of invertebrate parasites, and 5 families of vertebrate parasites (Blaxter & Koutsovoulos, 2014). Sudhaus (2008) suggests that there were independent events of insect parasitism acquisition in at least 20 lineages of the order Rhabditida. Indeed, different insect parasitism strategies have been reported, such as the family Mermithidae which includes obligatory parasite nematodes, or the family Phaenopsitylenchidae with facultative parasites. Additionally, the Steinernematidae and Heterorhabditidae families are both classified as strict parasites.

There are noteworthy phylogenetic associations between parasitic nematodes of invertebrates and those of vertebrates (Blaxter et al., 1998). For instance, the superfamily Strongylomorpha (of the suborder Rhabditina) encompasses parasites of the digestive tract and respiratory pathways of vertebrates and is phylogenetically linked to Heterorhabditidae. Similarly, Strongyloidoidae (a family of Rhabditida) comprises mammalian parasites that are related to the family Steinernematidae. These examples suggest that parasitism in vertebrates arose from entomopathogens (Blaxter & Koutsovoulos, 2014). These precedents make Nematoda an ideal phylum for studying the evolution and ecology of parasitism for two reasons: first, free‐living species related to parasitic ones share characteristics with ancestral precursors of parasitic lineages; second, the parallel evolution of parasitism allows for comparing different pathways to parasitic lifestyles and identifying common traits among them (Sommer & Ogawa, 2013). The evolution of parasitism can be understood by examining facultative parasites, which are also found in Nematoda. The ability of these organisms to alternate between free‐living and parasitic lifestyles represents an intermediate state in the evolution of parasitism. By exploring variation in life history, the level of adaptation of nematodes to either parasitic or free‐living lifestyles could be assessed. Nonetheless, it is crucial to consider the ecological and evolutionary factors that have driven the transition. In the next section, we will describe the general biology of entomopathogenic nematodes (EPN) and then, analyze the properties of rhabditids that favored the evolution of parasitism, especially of entomopathogeny. Therefore, we will examine similarities and differences of the archetypical EPN with the reported rhabditids to support the notion that this attribute has evolved in various families within Rhabditida. Finally, we will propose an ecological evolutionary concept of entomopathogenicity and underscore the importance of considering this approach in the study of the evolution of parasitism in the phylum Nematoda.

## 3 THE ENTOMOPATHOGENIC NEMATODES

**FIGURE 1:**
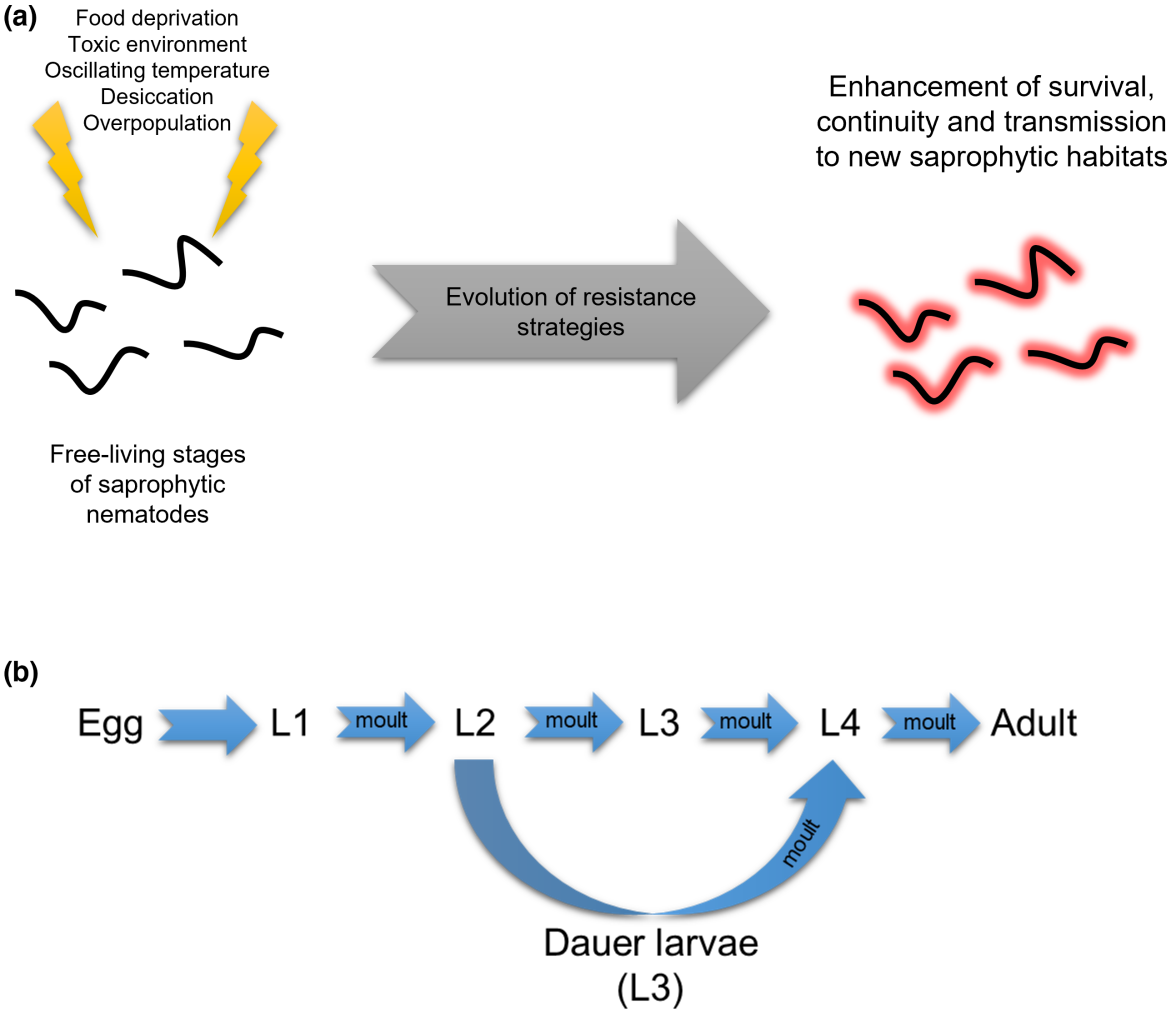
(a) The evolution and significance of resistance strategies in saprophytic nematodes. The free‐living stages of these nematodes face a range of environmental factors that impose significant selective pressures, leading to the development of resistance strategies. These protect, enhance survival, and promote long‐term transmission. (b) Basic life cycle of the rhabditid nematodes. The dauer larvae is an alternate form of the L3 larvae that remain in this stage until favorable conditions resume its development.

The symbiosis between EPN and bacteria is considered one of the most remarkable mutualistic relationships in the phylum (Box 2 in Appendix S1; Dillman et al., 2012). The species from the Steinernematidae and Heterorhabditidae families are associated with the Gram‐negative bacteria *Xenorhabdus* and *Photorhabdus*, respectively (Blaxter et al., 1998; Maher et al., 2017). These bacteria not only exhibit extreme virulence but also aid in the establishment and adaptation of their nematode hosts by defending insect corpses against predators, scavengers, and competitors, producing nutrients from the corpses, and promoting nematode development and reproduction. In turn, nematodes protect the bacteria from soil environmental hazards and transport them directly from the nutrient‐rich hemolymph of one insect to the hemolymph of another insect (Goodrich‐Blair & Clarke, 2007; Stock, 2015). EPN have a life cycle similar to that of other rhabditids, which includes the development of the dauer or infective juvenile stage (Figure 1b). This stage is free‐living and actively seeks out hosts to infect in the soil. It carries the symbiotic bacteria internally and, depending on the nematode genus, they will be located in different regions. The *Steinernema* species retains *Xenorhabdus* in a specialized region of the intestine within a special vesicle, while *Heterorhabditis* species harbors *Photorhabdus* in the anterior region of the intestine or along it (Chaston et al., 2011). The location of the insect host is possible through a series of foraging strategies of the dauer that range from immobile persistence to active searching (Lewis et al., 2006). Nematodes that actively search for hosts are called “cruisers.” The volatile chemical signals emitted by hosts stimulate these behaviors, which involve undulatory movements and even jumps (Campbell & Gaugler, 1997; Lewis et al., 1993). This strategy enables them to find sedentary hosts that remain immobile for long periods. However, when it comes to wandering hosts, nematodes called “ambushers” employ a strategy called nictation, a common behavior among phoretic nematodes. This ability consists of the elevation of the anterior region of the body and its subsequent waving movement from side to side, searching for potential hosts to attach to (Brown et al., 2011).

The mechanism of host entry varies depending on the nematode genus. For instance, *Steinernema*'s dauer larvae penetrates the insect's hemolymph through natural orifices, such as the mouth, anus, or spiracles. Larvae of *Heterorhabditis* feature an elongated tooth in the apical region, enabling them to pierce thin intermembrane regions of the host and gain direct access to the hemocoel (Koppenhöfer et al., 2007). Subsequently, within the hemocoel, the larvae transition from the dauer stage by shedding the extra L2 cuticle, resulting in the exposure of the mouth and anus. This process facilitates the expulsion of bacteria through regurgitation or defecation into the hemocoel (Ciche & Ensign, 2003). The nematode and its bacteria cooperate to overcome the host's immune response and kill it. Then, the bacteria biotransform the insect's tissues and grow on them, while the nematodes feed on the bacteria and process material for one to three generations inside the host's corpse. When the corpse is almost completely exploited, L2 larvae enter the dauer stage (L3) with their bacteria and seek a new host to infect. It is worth noting that *Steinernema* obtains its symbionts through trophic means, while *Heterorhabditis* transmits them maternally (Enright & Griffin, 2004; Poinar, 1990). These attributes serve as reference points to validate the authenticity of the entomopathogenic trait. However, this perspective disregards the context of evolutionary convergence. Consequently, entomopathogenicity can manifest through diverse mechanisms, not exclusively adhering to the currently recognized mode, especially when the ecologic evolutionary context of rhabditids is not considered. In the next section, we will explore the biological attributes within Rhabditida that lead to the evolution of parasitism, such as entomopathogeny.

## 4 THE TRANSITION TO PARASITISM IN NEMATODA

The evolution of parasitism in nematodes has been the subject of extensive research (Anderson, 1984; Osche, 1956; Poinar, 1983; Sommer & Ogawa, 2013; Sudhaus, 2010; Weischer & Brown, 2001), which has led to the development of an important conceptual framework: parasitism in nematodes originated from adaptations of saprophytic nematodes (see Figure 1a and Box 1 in Appendix S1; Osche, 1962, 1963). A comprehensive examination of the evolutionary and ecological factors associated with saprophytic nematodes offers insights into this transition. Various Rhabditida nematodes, such as *Caenorhabditis*, *Pristionchus*, *Rhabditis*, and *Oscheius*, are saprophytic and thrive in decomposing organic matter. The abiotic (and even biotic) conditions found in saprophytic environments favored adaptations that allowed nematodes to withstand and counteract them (Osche, 1956; Sudhaus, 2008, 2010), similar to what parasites encounter in live hosts (see Box 1 in Appendix S1; Dieterich & Sommer, 2009; Poulin, 2007; Sommer & Ogawa, 2013).

The interaction between saprophytic nematodes and bacteria was also a crucial selective pressure (Box 1 in Appendix S1), as bacterial exoenzymes or metabolites are toxic and limit the coexistence of other organisms. Rhabditids evolved a cuticle capable of neutralizing this toxicity (Ogawa & Sommer, 2009; Sudhaus, 2010), which eventually led to tolerate the digestive enzymes present in an invertebrate host's digestive tract (Sudhaus, 2010). Although bacteria serve as the main food source for rhabditids such as *Caenorhabditis*, *Mesorhabditis*, or *Rhabditis*, research indicates that the pathogenic activity against them varies among bacteria species. For example, *Caenorhabditis elegans* is susceptible to infections by *Staphylococcus aureus* and *Pseudomonas aeruginosa*, while *Pristionchus pacificus* is more resistant to their toxins, managing to feed on them and retaining some in its digestive tract (Rae et al., 2008). It is possible that tolerance to certain bacteria favored the colonization of specific saprophytic environments, such as dead insects or plants undergoing decomposition, and consequently, interaction with specific bacteria. On the other hand, the highly isolated and ephemeral distribution of saprophytic environments within ecosystems required the development of strategies that guarantee survival, reproduction, and dispersal (Ogawa & Sommer, 2009). Juvenile stages of saprophytic nematodes, such as the infective stages of parasitic nematodes, are free‐living and exposed to environmental threats. Therefore, nematodes evolved strategies that ensured persistence, time–space dispersal, and survival in case of food scarcity, low humidity, high temperatures, or high population density (Figure 1a).

Parasitic nematodes are characterized by the diversity of latency and resistance strategies that have evolved in response to common selective pressures, such as environmental adversities. For instance, certain plant parasites such as *Heterodera avenae*, the cereal nematode, survive dry seasons by forming cysts (Cooper & Van Gundy, 1971), while the eggs of *Meloidogyne* spp., the root‐knot nematodes, are protected by sacs (Cooper & Van Gundy, 1971). Also, the juvenile and adult stages of the garlic parasitic nematode *Ditylenchus dipsaci*, survive the summer by entering an anabiotic state in which they lose all the body's water (Cooper & Van Gundy, 1971; Norton, 1978). In contrast, when several families of Rhabditida, including Diplogasteridae, Rhabditidae, Heterorhabditidae, and Steinernematidae faced environmental stresses such as starvation, high population density, or heat, they promote the development of a juvenile stage called the dauer larvae (Riddle, 1988). Dauer is a German word that means “resistant” or “enduring,” and describes perfectly this evolutionary innovation that confers exceptional resistance to adverse soil conditions for extended periods of time, unlike the other life cycle stages (Figure 1b; O'Riordan & Burnell, 1990). This alternative form of the third stage juvenile larvae (L3) retains the L2 cuticle and is encased in a new cuticle that extends beyond the mouth and anus, providing special protection. The dauer larvae does not feed and uses lipid and glycogen reserves as its primary energy sources (Burnell et al., 2005), while remaining active in search of a host. Studies examining the survival of the dauer larvae in *C. elegans* and some entomopathogens, such as *H. bacteriophora*, have shown that it can survive in soil for up to 3 months (Grewal et al., 2002; Hu, 2007). Therefore, this stage represents a critical adaptation to saprophytic life and is of significant fitness value, contributing to the spatiotemporal dispersal of EPN. The transition from L2 to dauer larvae involves significant anatomical modifications, such as a reduction of the intestine, inactivation of excretory glands, and modification of the amphids (sensory organs) that act as chemoreceptors (Grewal et al., 2002; Riddle, 1988). These modifications have resulted in the development of specific behavioral traits and recognition mechanisms for insects and other invertebrates in the environment (Lewis et al., 1992; Poinar, 1983; Poulin, 2007; Sudhaus, 2010). Therefore, the conserved morphological and physiological similarity between the dauer larvae of saprophytic nematodes and the infective juvenile (IJ) or “infective juvenile larvae” of EPN makes it a valuable subject of study in parasitology, as it is the stage that locates and infects the insect host (Lewis et al., 1992). In the following section, we will provide a detailed explanation of the different interactions between nematodes and insects, with the dauer larvae playing a critical role in each interaction and the evolution of the entomopathogenic trait.

## 5 NEMATODE–INSECT ASSOCIATIONS AS DRIVERS OF ENTOMOPATHOGENICITY

**FIGURE 2:**
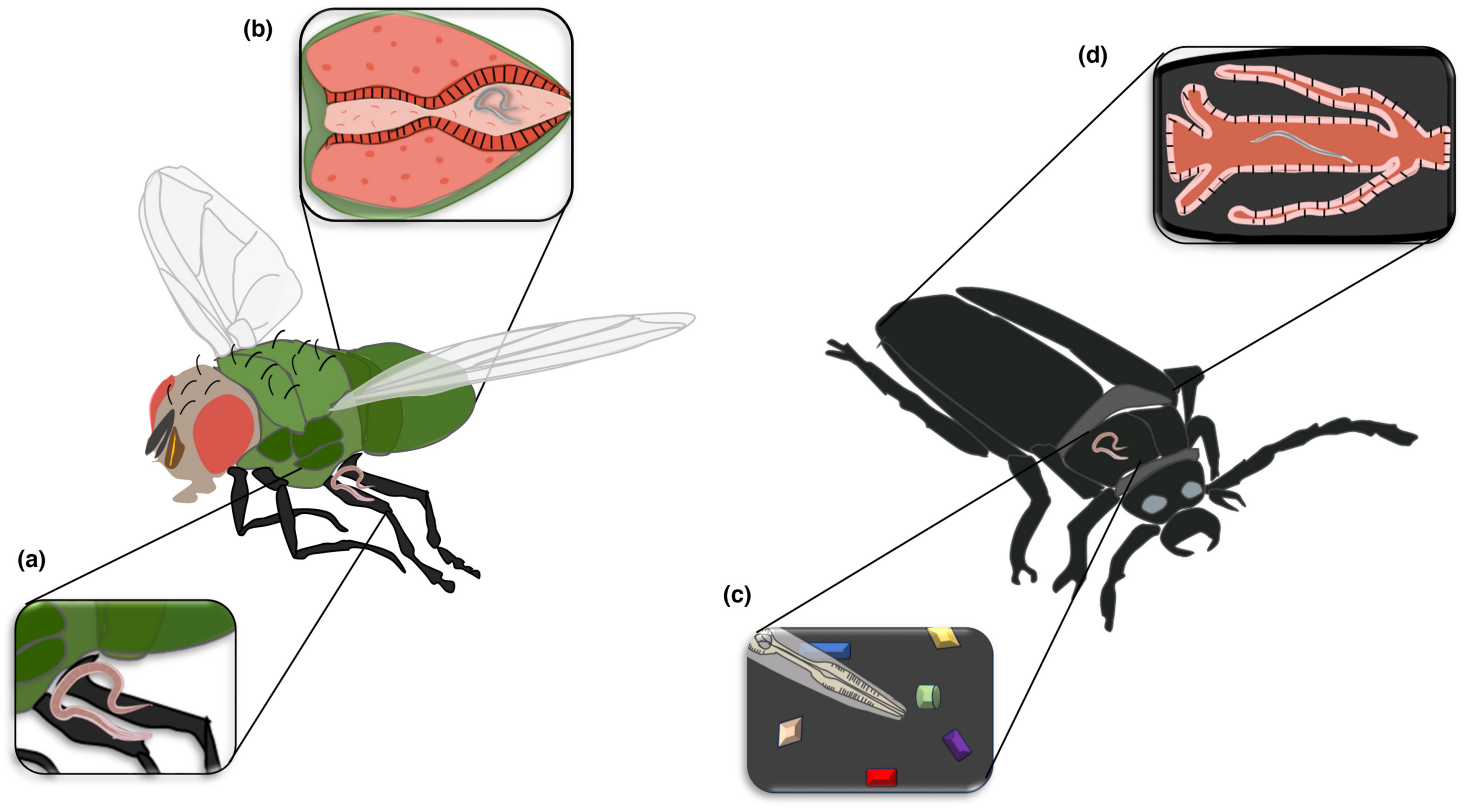
Nematode–insect associations. (a) Phoresis: Dauer larvae are transported by insects among environments; (b) Endophoresis: Dauer larvae are transported internally by the insect; or Necromenia: Dauer larvae remain within the insects until they naturally die to resume its development; (c) Entoecy: Dauer larvae resume its development and feed on bacteria or fungi found on insect's cuticle; (d) Parasitism: Dauer larvae resume its development internally and feed on insect's tissues and resources.

Nematodes and insects are both ancient groups, with the first lineages of insect parasitic nematodes possibly originated in the late Devonian period (~ 400 million years ago) (Meldal et al., 2007; Sudhaus & Fitch, 2001). The associations between these organisms can be either commensal or antagonistic (Table 1, Figure 2; Gaugler & Kaya, 1990), and is proposed that parasitism evolved from commensal interactions (Dieterich & Sommer, 2009; Osche, 1956; Poulin, 2007). This is because associations exist within a continuous threshold of possibilities linked by intermediaries, allowing one type of interaction to potentially evolve into another (see Box 1 in Appendix S1; Maggenti, 1981; Sudhaus, 2008). Below, we will review the strategies used by nematodes to survive. These strategies are also summarized in Table 1 and illustrated in Figure 2, with examples of each one in Table 2.

**TABLE 1:** Types of nematode–insect associations.

Commensal	Antagonist
*Phoresis* The use of insects or another invertebrate as transport to new habitats. The dauer larvae is the specialized phoretic stage of the saprophytic nematodes (Poinar, 1983) *Endophoresis* Also called “internal phoresis,” refer to the nematode's ability of enter the host without harming it and gaining an additional protection (Sudhaus, 2010) *Entoecy* The nematode invade host's cavities and feed on the bacteria and fungus founded there to complete its life cycle. This does not harm the host (apparently) (Sudhaus, 2008) *Necromeny* This is a Greek term that means “wait inside the body until the decay of the corpse.” When the host dies by natural causes, the dauer larvae resume its development feeding on the bacteria that multiply on the carcass (Sudhaus & Schulte, 1989)	*Parasitism* The parasites obtain nutrients directly from the host to complete its life cycle. Commonly, the parasite nematodes invade the digestive tract, Malpighi tubules or the hemolymph (Poinar, 1972) *Entomopathogeny* Cooperation between nematodes and entomopathogenic bacteria. Inside the insect, the dauer larvae or infective juvenile sheds its cuticle to release the bacteria. Then both organisms cooperate to overcome the host's immune response, killing it by sepsis (Eleftherianos et al., 2018; Forst & Clark, 2002). The bacteria biotransform the carcass and the nematodes feed on them and the converted tissues (Gaugler, 2002)

**TABLE 2:** Examples of the different nematode–insect associations other than entomopathogenicity.

Association	Species	Host	Reference
Phoresis			
	*Pelodera coarctata*	*Aphodius*	Sudhaus (1976)
	*Choriorhabditis dubia*	*Psychoda* sp.	Sudhaus and Kühne (1990)
	*Turbatrix aceti*	*Drosophila* sp.	Clark (1994)
Endophoresis			
	*Rhabditoides stammeri*	*Nicrophorus*	Richter (1993)
	*Caenorhabditis drosophilae*	*Drosophila nigrospiracula*	Kiontke (1997), Kiontke and Sudhaus (2006)
	*Koemeria hirschmannae*	*Geotrupes*	Kanzaki (2017)
	*Paroigolaimella coprophila*	*Sepsis*	Kiontke (1996)
	*Rhabpanus ossiculum*	*Reticulitermes flavipes*	Massey (1971)
	*Oigolaimella attenuata*	*Reticulitermes* spp.	Fürst von Lieven and Sudhaus (2008)
	*Diploscapter lycostoma*	*Linepithema humile*	Wahab (1962)
	*Oscheius dolichurus*		
	*Koemeria histophora*		
Entoecy			
	*Rhabditis adenobia*	*Oryctes* spp.	Poinar (1971)
	*Crustorhabditis*	*Ocypode* spp.	Sudhaus (1976)
Necromeny			
	*Oscheius necromenus*	Myriapods	Schulte (1990)
	*Diplogasteroides nasuensis*	*Melolontha hippocastani*	Manegold and Kiontke (2001)
	*Oscheius insectivorus*	Coleopterans	Kiontke and Sudhaus (2006)
Parasitism			
	*Diploscapter lycostoma*	*Iridomyrmex humilis*	Markin and McCoy (1968)
	*Parasitorhabditis* spp.	*Orthotomicus proximus*	Poinar (1972)
	*Oscheius dolichurus*	*Lasius brunneu*	Köhler (2012)
	*Rhabpanus ossiculum*	*Reticulitermes flavipes*	Massey (1971)
	*Diplogastrellus secundus*	Aphodius	Bovien (1937)
	*Monochoides aphodii*	Aphodius	Poinar et al. (1976)

The initial association between these organisms is referred to as “phoresy” and marks a pivotal moment in the evolutionary history of nematodes as it introduced novel selective pressures that led to specialized adaptations against insects. As a result, the dauer larvae evolved the ability to respond to chemical and mechanical cues to identify and adhere to insects, frequently in saprophytic environments, such as beetles and some dipterans (Kiontke, 1999; Rühm, 1956; Sudhaus, 1976; Warburton & Zelmer, 2010). The sensory and neurological features of these larvae are exaptations associated with the evolution of an infectious parasitic stage that requires mechanisms to identify and select a host. Its chemotactic ability has been demonstrated using insect sex pheromones, insect semiochemicals, and plant semiochemicals. Also, studies have revealed unique chemotaxis profiles for *Pristionchus* spp. (Herrmann et al., 2006; Hong et al., 2008; Hong & Sommer, 2006). For instance, *P. pacificus* exhibits strong and specific attraction to the sex pheromone ((Z)‐7‐tetradecen‐2‐one, ZTDO) of *Exomala orientalis* (Coleoptera) (Herrmann et al., 2007), while *P. maupasi* is attracted to phenol, a sexual attractant of the dung beetle, whereas other *Pristionchus* species show no attraction to this compound (Hong et al., 2008). Although current evidence suggests that nematodes use host sexual communication pathways to locate them, it would be interesting to investigate whether these pathways are common in finding hosts in Nematoda, whether they have evolved in association with EPN, or whether there are multiple independent pathways for host recognition. Phoresis provides an advantage for nematodes, as they use the host as transportation, dispersion, and/or shelter without compromising its fitness (Houck, 2009; Signe‐White et al., 2017). However, if the nematode enters the host to travel (i.e., endophoresis), this implies a different environment and lifestyle that can impact behavioral aspects of the dauer larvae (Sudhaus, 2008). This exaptation gave rise to other commensal associations such as necromeny, where the larvae remain in the dauer stage inside the host (see Table 1; Blanco‐Pérez et al., 2017; Sudhaus & Schulte, 1989). Living inside the host exposed nematodes to new selective pressures, resulting in antagonistic interactions similar to entomopathogenicity and parasitism that significantly affect the host's fitness (Sudhaus, 2008). Both nonbacterial‐mediated parasitic interaction and entomopathogenicity are facilitated by the benefits associated with exploiting the host's resources, such as the insect hemolymph, which is an excellent source of nutrients for nematodes due to its high levels of the disaccharide trehalose (Dahlman & Vinson, 1976; Sudhaus, 2008). However, these associations have differential impacts on the host. Parasites obtain resources from the host without killing it or damaging its reproductive potential, as their progeny is generated and transmitted from the living host. The death of the host would therefore incur a significant evolutionary cost in the adaptation of the parasite. In contrast, entomopathogens kill the host to ensure the resource acquisition and transmission of their progeny (Table 1; Brivio & Mastore, 2018; Ebert & Weisser, 1997). In this context, it is pertinent to highlight that nematodes belonging to the Mermithidae family are deemed entomopathogens, akin to EPN, for their capacity to kill insects as part of their life cycle (Georgis et al., 2006; Sepulveda‐Cano et al., 2008). Nevertheless, the underlying mechanisms are markedly divergent. Mermithids infect at larval stage L2 and absorb nutrients from the insect through their cuticle, a phase recognized as the parasitic stage. However, upon reaching the L3 stage, they go out to the host, resulting in its demise (akin to parasitoids), and complete their maturation in aquatic soils (Federici, 1999). Contrary, EPN employ entomopathogenic bacteria to kill the host starting from their L3 stage, utilizing the deceased host as nourishment for both the nematode's growth and its successive generations (Lewis & Clarke, 2012). With these distinctions in mind, it is crucial to recognize the varied parasitic strategies employed by these organisms and to bear that the term “pathogen” is conventionally applied to microparasites (e.g., viruses or bacteria). In this instance, EPN are classified as such owing to their interaction with bacteria, in contrast to mermithids.

Unlike necromeny, entomopathogenicity involves the nematode “carrying” its primary food source to the host and using it to kill it (Sommer & Ogawa, 2013; Sudhaus, 2010). Some studies suggest that necromeny precedes entomopathogenicity, as demonstrated in the *Oscheius necromenus* system and the millipede *Ommatoiulus moreletii* (Schulte, 1990). When the dauer larva of *O. necromenus* penetrates the gut wall of the millipede *O. moreletii*, bacteria (Enterobacteriaceae) adhere to its cuticle and invade the insect's hemocoel, causing its death. This undoubtedly activates the millipede's recognition and immune response mechanisms, which in turn activate both bacterial and nematode immune evasion mechanisms. In this context, the host's immune response is an important selective pressure for the evolution of entomopathogenicity. The specificity and preference of nematodes for certain bacteria lead to an alternate evolutionary pathway: mutualism that confers entomopathogenic traits. In Nematoda, only the *Steinernema*‐*Xenorhabdus* and *Heterorhabditis*‐*Photorhabdus* systems are recognized as true entomopathogenic associations (Dillman et al., 2012). Nevertheless, nematodes from other families, such as Rhabditidae and Diplogasteridae, have been found to associate with other entomopathogenic bacteria genera, such as *Serratia* or *Bacillus* (Ortega‐Estrada et al., 2012). This suggests the existence of additional entomopathogenic lineages beyond *Steinernema* and *Heterorhabditis*.

## 6 ACTUAL REQUIREMENTS TO CONSIDER NEMATODES AS “ENTOMOPATHOGENIC NEMATODES”

The appropriate criteria for defining an EPN are primarily grounded in the distinguished traits of the representative families. Undoubtedly, Steinernematidae and Heterorhabditidae have demonstrated an extraordinary mutualistic bond with their bacteria, resulting in all described species within these families being EPN. Nevertheless, evolution also leads to evolutionary analogies. Often, when new associations of nematodes with entomopathogenic bacteria are reported, they are judged based on a series of “distinctive traits” (Chaston & Goodrich‐Blair, 2010; Dillman et al., 2012; Rae & Sommer, 2011):

1. The dauer larva (i.e., infective juvenile stage) is the only stage with a free lifestyle in soil.
2. The dauer larva seeks for a host and penetrating it.
3. Only the dauer larva can harbor pathogenic bacteria as symbionts.
4. Symbiotic bacteria are released into the insect's hemocoel, promoting rapid host death (i.e., <120 h).
5. The bacteria biotransform the host cadaver, contributing to their proliferation and the nematode's development.
6. The reproduction of nematodes depends on their symbiotic bacteria.
7. The L2 larvae from the last generations recruit their symbiotic bacteria and develop into the dauer stage they leave the cadaver and search for new hosts to infect.

Considering that there are more than 70 known species of *Steinernema* and 18 of *Heterorhabditis*, these requirements are based on less than 5% of these species (Adams et al., 2006; Murfin et al., 2012). Therefore, these features cannot be considered distinctive of the nematode entomopathogenity. In biological control, the interest in EPN is limited to their virulence, searching for the “ideal” nematode to combat pests. However, this approach overlooks the interaction with the host, including their interaction with the immune response of insects (Brivio & Mastore, 2018; Georgis et al., 2006; Laznik & Trdan, 2014). This is of paramount importance as neutralizing this potential threat is crucial for ensuring the effectiveness of biological control. Therefore, it is imperative to take into account that the immune response of insects displays variations that can potentially interfere with the immunomodulatory capabilities of EPN (Schmid‐Hempel, 2005). An illustrative instance is evident in *Galleria mellonella*, where its hemocytes can identify the dauer larvae of *H. bacteriophora* yet fail to recognize those of *S. carpocapsae* and *S. glaseri* (Ebrahimi et al., 2011). On the other hand, the possibility that the nematode is only infective in a particular stage of its host or has plasticity of parasitism, transitioning between free‐living and infective phases, is not considered. Incorporating fundamental biological aspects such as interaction, virulence plasticity, behavior, lifestyle, and natural habitats will facilitate the inclusion of diverse nematode species in a general concept of parasitism for the phylum, leading to the development of a robust and inclusive theoretical framework.

## 7 THE ENTOMOPATHOGENICITY IN OTHER RHABDITIDS

In recent years, several species of the genera *Caenorhabditis*, *Pristionchus*, *Oscheius*, and *Rhabditis* have been reported to be associated with entomopathogenic bacteria, either in ectosymbiosis or endosymbiosis (see Table 3; Abebe et al., 2010; Clarke & Eberl, 2006; Dillman et al., 2012; Jiménez‐Cortés et al., 2016; Shan et al., 2019; Zhou et al., 2017). Although these nematodes have been experimentally demonstrated to possess the ability to locate and kill host insects, fundamental details of the experimental methods are missing or not tested for some of them, which raises doubts about their entomopathogenic nature. For instance, the identity of the bacteria and their location within the nematode remains unknown for species such as *O. amsactae*, *O. basothovii*, *O. gingeri*, *O. niazii*, *O. onirici*, and *O. siddiqii*. This information is essential for understanding the nature of symbiosis, including its persistence (temporal/long‐term), type (ectosymbiosis/endosymbiosis), and whether the parasitism is facultative or obligate (Abebe‐Akele et al., 2015; Boemare et al., 1997; Goodrich‐Blair, 2007; Murfin et al., 2012). Among the reported rhabditids, only two have an ectosymbiotic association: *O. caroliniensis* and *P. entomophagus* (as shown in Table 3). The nature of these symbioses can be explained by their lifestyle. *Oscheius* species are free‐living, saprophytic, phoretic, and necromenic, which are also properties shared by species of *Pristionchus*, another genus of the family Diplogasteridae (Ali et al., 2011; Rae et al., 2008; Ragsdale et al., 2015). Saprophytic environments are ideal for the proliferation of potentially pathogenic bacteria, such as *Serratia* species. And in a necromenic association, after the host's death, a process of colonization by bacteria and fungi begins, turning the host into a toxic “insect soup,” with the nematode and numerous microorganisms competing for the resources offered by the corpse. So, the development, reproduction, and emergence of progeny in the form of dauer larvae (from the insect corpse or a saprophytic habitat) is a viable option for opportunistic bacteria and fungi capable of adhering to them and thus being transported to nutrient‐rich habitats such as hemolymph (Blanco‐Pérez et al., 2017; Rae et al., 2008). This interaction meets the profile of phoresis, which mainly favors the bacteria's fitness. However, from an ecological evolutionary perspective, the antagonism of nematodes with insects suggests the evolution of a mutualistic link, where both interactants obtain benefits derived from successful parasitism in insects from reciprocal adaptations (Thompson, 1982). An authentic ectosymbiotic link can be defined by the capacity for permanence and specificity of the bacteria on the nematodes' cuticles. In this sense, the easy removal of *S. marcescens* from the cuticle of *O. caroliniensis* and its absence in nematodes of the same species within the collection area suggest that it is a facultative and short‐term ectosymbiosis (Torres‐Barragan et al., 2011), similar to what is observed in *Phasmarhabditis hermaphrodita*, a rhabditid that infects snails and forms weak associations with bacteria (Rae et al., 2010). Conversely, molecular studies in *Paenibacillus nematophilus* revealed its specificity for the cuticle of species of *Heterorhabditis*, based on the interaction of specific oligosaccharides with lectins conserved in the cuticle. In addition, this species shows tolerance to the antibiotics generated by the symbiont *Photorhabdus* sp. (Enright & Griffin, 2004). Specific ectosymbiosis of *S. marcescens* with *Steinernema* has also been documented, and the coexistence of this bacterium with the natural symbiont *Xenorhabdus* sp. in the host's corpse indicates tolerance to its antibiotics (Ortega‐Estrada et al., 2012).

**TABLE 3:** List of entomopathogenic rhabditids.

Genus	Species	Entomopathogenic bacteria	Other bacteria	Location in the host	Collected from	Reference
*Caenorhabditis*	*Briggsae*	*Serratia* sp. SCBI	ND	Gut	*Galleria mellonella* White trap	Abebe et al. (2010)
*Heterorhabditoides*	*Chongmingensis*	*Serratia nematodiphila*	ND	Gut	*G. mellonella* White trap	Zhang et al. (2008)
	*Rugaoensis*	*Serratia nematodiphila*	ND	Gut	*G. mellonella* White trap	Zhang et al. (2012)
*Oscheius*	*Amsactae*	ND	ND	ND	*Amsacta moori* (Lepidoptera) larvae	Ali et al. (2011)
	*Basothovii*	ND	ND	ND	*G. mellonella* White trap	Lephoto and Gray (2019)
	*Caroliniensis*	*Serratia marcescens*	ND	Cuticle	*G. mellonella* White trap	Torres‐Barragán et al. (2010)
	*Gingeri*	ND	ND	ND	*Zingiber officinale* Rosc. roots	Pervez et al. (2013)
	*Microvilli*	Serratia sp.	ND	Gut	*G. mellonella* White trap	Zhou et al. (2017)
	*Niazii*	ND	ND	ND	Palm roots	Tabassum and Shahina (2010)
	*Onirici*	ND	ND	ND	*G. mellonella* White trap	Torrini et al. (2015)
	*Siddiqii*	ND	ND	ND	Bushes roots	Tabassum and Shahina (2010)
*Pristionchus*	*Entomophagus*	*Serratia marcescens* *Serratia nematodiphila*	*Pseudomonas fluorescens*	Cuticle and Gut	*Myrmica rubra* (Hymenoptera)	Ishaq et al. (2021), Michaud (2013)
	*Entomophilus*	*Serratia* sp.	ND	Gut	*G. mellonella* White trap	Li et al. (2015)
*Rhabditis*	*Blumi*	*Providencia vermicola* *Flavobacterium* sp. *Alcaligenes faecalis*	ND	Gut	*Exomala orientalis* (Coleoptera)	Park et al. (2011)
	*Regina*	*Alcaligenes faecalis* *Actinomyces* sp. *Enterobacter ludwigii* *Klebsiella ocytoca* *Serratia marcescens* *Bacillus thuringensis*	*Pseudochrobactrum* sp. *Bordetella bronchiseptica* *Citrobacter freundii* *Pseudomonas nitroreducens* *Leucobacter* sp. *Gordonia* sp.	Gut	*P. polyphylla*, *Paranomala* sp. and *Cyclocephala* sp. larvae	Jiménez‐Cortés et al. (2016)

*Note*: The list is shown by the genera and species of reported nematodes, the associated bacteria, their location in the nematode host, and the insect host from which it was collected. For the search of the mentioned species, the following terms were used: “entomopathogen nematode”, “entomopathogenic nematode”, “entomopathogen rhabditid” “entomopathogenic rhabditid”, “insect pathogenic nematode”, “insect pathogenic nematode‐bacteria complex”, “insect pathogenic nematode‐bacteria symbiosis” and “pathogenic nematode‐bacteria symbiosis”. The search engines used were The Web of Science, Scopus, Ovid, Agris, ProQuest Biological Sciences, and Google Scholar.

Abbreviation: ND, not determined.

While not regarded as authentic entomopathogens, the ectosymbiotic associations of *O. caroliniensis* and *P. entomophagus* are noteworthy and informative because they represent intermediate stages in the evolutionary continuum of interactions among nematodes, bacteria, and insects (Box 1 in Appendix S1). It is worth emphasizing that these associations enable an alternative lifestyle. Depending on the bacterial species they associate with, the nematodes can either function as entomopathogens or exist as free‐living organisms. The association can also be endosymbiotic, suggesting a more intimate link as it involves nematode *selectivity* for the bacteria it ingests (Shapira, 2017). So, the bacteriophagy can be specific or nonspecific (Freyth et al., 2010; Murfin et al., 2019). Rhabditids prefer feeding on Gram‐negative bacteria due to their thin cell walls that are easily digested compared to Gram‐positive bacteria (Salinas et al., 2007; Tortora et al., 2000). For instance, *C. elegans* prefers Gram‐negative bacteria that benefit its development and reproduction (Artan et al., 2016). Nematodes recognize beneficial bacterial genera or species that are preferentially ingested or specific (Goodrich‐Blair, 2007; Ott et al., 1991). In this regard, *Heterorhabditis* and *Steinernema* species are highly selective with their symbiotic bacteria (Bird & Akhurst, 1983; Gerritsen & Smits, 1997). When these nematodes are cultured with nonsymbiotic bacteria in vitro, they can distinguish them from their original symbionts, resulting in monogenic microbiotas within dauer larvae (Ciche et al., 2006).

Phylogenetic analyses support the hypothesis that the entomopathogenic behavior of Heterorhabditidae and Steinernematidae originated from the ancestral trophic behavior of free‐living bacteriophagous nematodes (Box 2 in Appendix S1; Blaxter et al., 1998; Poinar, 1993). Within Rhabditida, there are morphological adaptations related to foraging. For instance, genera such as *Caenorhabditis*, *Heterorhabditidoides*, and *Rhabditis* from the family Rhabditidae possess a grinding apparatus in their esophagus to break down the bacteria they ingest; however, some of these bacteria manage to “escape” and remain viable in the digestive tract (Aballay et al., 2003; Labrousse et al., 2000). In contrast, the genus *Pristionchus* from the family Diplogasteridae lacks this “grinder,” causing some of the ingested bacteria to remain intact while others undergo lysis (Chiang et al., 2006; Fürst von Lieven & Sudhaus, 2000). The idea that rhabditids acquire bacteria in saprophytic environments or inside insects (via necromeny) suggests that their chances of interacting with entomopathogenic bacteria are higher. For example, bacteria of the genus *Serratia* (Enterobacteriaceae) are abundant in soil and in the digestive tract of insects, several of which are entomopathogenic and associated with different rhabditids such as *C. briggsae*, *Heterorhabditidoides chongmingensis*, *H. rugaoensis*, *O. microvilli*, and *P. entomophilus* (Table 3). Future research should explore nematode‐ and bacteria‐related factors, including genetics, immunology, physiology, and ecology, that either facilitate or impede the colonization of the rhabditids' digestive tract. Investigating these aspects is crucial for delineating the boundaries of nematode–bacteria endosymbiotic associations and understanding the selective pressures that influence and mold them.

A paradigm exists regarding the seemingly monogenic microbiome of entomopathogenic nematodes (Box 3 in Appendix S1). Studies are often focus on bacteria responsible for conferring the entomopathogenic character while the microbiota is far more diverse. It is worth noting that the Enterobacteriaceae group is abundant in diverse soil habitats, and numerous Proteobacteria exhibit a tendency to form associations with nematodes, endowing them with unique biological characteristics (Table 4; Boemare et al., 1997; Gouge & Snyder, 2006; Koppenhöfer, 2007; Ortega‐Estrada et al., 2012; Sajnaga & Kazimierczak, 2020). However, few studies have extensively examined the microbiota of rhabditids, such as *Rhabditis blumi* and *R. regina*. In them, strains of *Serratia* spp., aquatic bacteria (Acinetobacter), rhizobacteria (i.e., *Pseudomonas nitroreducens*), and phytopathogenic bacteria like *Citrobacter* are found (Table 3; Jiménez‐Cortés et al., 2016; Tambong, 2013). Specifically, the ability of γ‐proteobacteria to establish symbiosis with rhabditids appears to have arisen multiple times throughout the evolutionary history of these bacteria (Sajnaga & Kazimierczak, 2020). The genera *Xenorhabdus* and *Photorhabdus*, which belong to this lineage, express similar attributes through convergent evolution (Box 2 in Appendix S1; Dillman et al., 2012; Husnik et al., 2011). The establishment and colonization of bacteria in the digestive tract of rhabditids have been explained by various hypotheses (Box 4 in Appendix S1). Results from experiments with *Escherichia coli* and *C. elegans* suggest that the persistence of bacteria in the nematode may be due to selection events occurring in the digestive tract, such as the evasion of antimicrobial defenses, obtaining or collecting nutrients, or the ability to adhere to the walls of the tract (Portal‐Celhay & Blaser, 2012). Below, we will show that entomopathogenic nematodes are not a black or white strategy but there are gray in between.

**TABLE 4:** Examples of the proteobacteria–nematode complexes according to their location (endo or ecto) and the potential function of bacteria for the nematodes.

Bacteria	Nematode	Location	Function	Reference
Sulfur‐oxidizing γ‐proteobacteria	*Laxus oneistus*	Ecto	Supply of primary nutrients of chemosynthesis	Bulgheresi (2011)
*Wolbachia pipientis*	*Onchocerca flexuosa* *Wuchereria bancrofti* *Loa loa*	Endo	Essential compounds synthesis: hemes groups, flavin adenine dinucleotide (FAD), riboflavins, glutathione, purins, pyrimidines, and nucleotides	Bouchery et al. (2013)
*Xiphinematobacter* spp.	*Xiphinema* spp.	Endo	Unknown. Its transmission is vertical	Mobasseri et al. (2019)
*Rhizobium* spp.	*Osheius tipulae* *Mesorhabditis* sp.	Endo	Colonization in roots	Matus‐Acuña et al. (2018)
*Xenorhabdus* sp.	*Steinernema* spp.	Endo	Entomopathogenic and supply of essential nutrients	Goodrich‐Blair and Clarke (2007)
*Photorhabdus* sp.	*Heterorhabditis* spp.	Endo	Entomopathogenic and supply of essential nutrients	Stock (2015)

## 8 REDEFINING ENTOMOPATHOGENICITY IN NEMATODA

The entomopathogenic trait has a polyphyletic origin within the order Rhabditida, manifested also in the families Diplogasteridae and Rhabditidae. While the evolutionary histories of these families are independent, their interactions with insects and bacteria have brought them together along an evolutionary continuum, represented by a range of lifestyles (Box 2 in Appendix S1). However, the associations reported for species of Diplogasteridae and Rhabditidae appear feeble when compared to those of Steinernematidae and Heterorhabditidae. For instance, nematodes such as *C. briggsae* or *R. regina* would not qualify as strict “entomopathogenic” under current criteria because the nature of these nematode–bacteria symbioses does not match with the archetypical EPN species. One argument against this perspective asserts that certain rhabditids lack morphological adaptations essential for a successful symbiosis with bacteria. This holds true, particularly for Steinernema species, which exhibit a bacterial receptacle to host the *Xenorhabdus* symbiont. In contrast, *Heterorhabditis* species lack this specific trait, yet the symbiont persists throughout their digestive tract. Notably, this location is shared with many other nematode species, including *Caenorhabditis*, *Pristionchus*, *Oscheius*, and *Rhabditis* (CITSS). Molecular studies have identified genes such as nilA, nilB, and nilC in *X. nematophila*, enabling its colonization within *S. carpocapsae* by encoding cell wall proteins that interact with the receptacle epithelium. This convergence of bacterial traits favoring intestinal colonization is not exclusive to *Xenorhabdus* or *Photorhabdus* but extends to other γ‐proteobacteria (Cowles & Goodrich‐Blair, 2008; Heungens et al., 2002), suggesting that successful symbioses with nematodes can occur with a diverse range of bacterial species. However, the skepticism toward non‐archetypical Entomopathogenic nematodes (EPN) stems from the concept of EPN possessing a “monogenic microbiota” (Box 3 in Appendix S1). Undoubtedly, the fitness of both *Steinernema* and *Heterorhabditis* depends on their bacterial mutualists (McMullen et al., 2017). Yet, many studies on EPN microbiota focus primarily on principal symbionts, neglecting the associated bacterial community and its functions. Ogier et al. (2020) propose that *Sterinernema*'s virulence results from the interaction of *Xenorhabdus* with other entomopathogenic bacteria, such as *Pseudomonas protegens* and *P. chlororaphis*, forming a “pathobiome.” This underscores the idea that nematode fitness relies on the synergy of its microbiota, with some members contributing to entomopathogenic attributes while others influence metabolic and immunologic capacities, as observed in other rhabditids. Therefore, the generalization of microbiota and the absence of specific bacteria in reported rhabditids do not negate their authentic entomopathogenic nature. It is crucial to acknowledge that *Steinernema* and *Heterorhabditis* may represent one extreme of the entomopathogeny spectrum, with specialized bacterial genera (*Photorhabdus* and *Xenorhabdus*), while other rhabditids follow alternative paths, sharing entomopathogenic bacteria genera like *Bacillus* or *Serratia*. The saprophytic habit of certain rhabditids, such as *Oscheius* or *Rhabditis* species, suggests a “facultative parasite or facultative EPN” nature. Nevertheless, while saprophytic ability has been demonstrated in laboratory conditions, lifestyle preferences are largely unexplored, and optimal foraging theory application could elucidate whether the entomopathogenic habit is preferred or merely an alternative. It is essential to note that EPN, despite having a saprophytic habit, are primarily considered to display an entomopathogenic lifestyle. However, gaps in EPN ecology knowledge and challenges in facing host temporality may occasionally lead EPN to exhibit their ancestral saprophytic habit. This nuanced perspective acknowledges that parasitism is not an all‐or‐nothing strategy; instead, facultative parasitism is also predicted.

Instead of redefining the term EPN, there is the concept of entomopathogenicity in Nematoda that needs to be clarified. Entomopathogenicity is a trait that appears in different families of Nematoda through convergent evolution, represented by the association between nematodes and entomopathogenic bacteria with independent evolutionary histories (Ciche et al., 2006; Murfin et al., 2012). The concept of coevolutionary mutualism suggests that such a relationship emerges based on various biotic and abiotic factors, as well as the continuity of interaction over evolutionary time and space (Thompson, 1982). Furthermore, for a successful alliance, three essential conditions must be met: (1) Mutual tolerance, where both entities can coexist without eliminating each other, as one might eventually displace the other; (2) Cost–benefit trade‐offs, wherein the advantages of living together outweigh the associated costs; without a positive balance, symbiosis may not be favored, potentially leading to one species abandoning the relationship or attacking the other; (3) Reproduction without compromise, allowing organisms to reproduce continuously without hindering each other's reproduction (Abebe et al., 2011; Roughgarden, 1983; Stearns, 1989). However, even though reported instances of rhabditids associating with and retaining bacteria without fatal consequences fulfill the first condition, the outcome of such interactions may not necessarily be mutualistic. This is because symbionts, at times, act in their selfish interests. Studies on coevolution between hosts and symbionts have demonstrated instances where symbionts enhance one host trait at the expense of another (Rudgers et al., 2012). For example, the improvement of *C. elegans* heat stress resistance by *B. subtilis* coincides with a reduction in its fecundity, resulting in an overall reduction in host fitness compared to hosts that do not coevolve with the symbiont (Hoang et al., 2022). This illustrates that coevolution does not necessarily lead to mutually beneficial associations (Moran & Sloan, 2015), and the evolution of mutualism can be constrained by emerging conflicts. Hence, is evident that the bacterial symbioses within Rhabditidae and Diplogasteridae deserve further studies to clarify the nature of the interactions. For instance, a comprehensive approach involves quantifying the costs and benefits associated with nematodes that have entomopathogenic bacteria (e.g., Serratia or Bacillus) versus those without, allowing for a direct comparison of the fitness of both partners. In essence, entomopathogeny is an ecological and evolutionary term encapsulating the nematode's ability to infect, kill, and feed on insects, aided by symbiotic bacteria. Specialized EPN share several biological attributes with “facultative entomopathogenic rhabditids.” Despite some disparities, such as specialized microbiota, the emergence of entomopathogeny within Rhabditida is evident through evolutionary convergence. The ecological and evolutionary histories of each group have unfolded in diverse scenarios, serving as a reminder that parasitic strategies, including entomopathogeny, can manifest in varied designs.

## 9 THE IMPORTANCE OF MICROHABITAT ON THE NEMATODE–BACTERIA SYMBIOSES

The heterogeneity of the environment is a critical factor contributing to the composition of nematode's microbiota (Berg et al., 2016). Soil is the primary habitat for rhabditids, where they interact with a diverse range of micro and macro faunas (Kiontke & Fitch, 2013). The diversity and richness of the bacterial communities in soil offer diverse ecto‐ and/or endosymbiotic associations. Notably, nematode bacteriophagy plays a crucial role in nutrient flow in the soil, making nematodes “bacteria disseminators” (Ekschmitt et al., 1999; Eller & Frenzel, 2001). But also, some of the transported bacteria may be pathogenic to plants and animals, further emphasizing their ecological significance. In addition to soil, novel associations may arise when bacteria are acquired almost exclusively from saprophytic media or inside the insects. The nature of these associations is contingent upon microhabitat biotic and abiotic factors, such as the composition of the bacterial community, the presence of competitors (including other bacteriophage nematodes), as well as temperature and moisture levels. However, while the entomopathogenic capacity of Rhabditidae may differ between populations, this field remains largely unexplored. For instance, a strain of *O. oniciri* collected from Italy expressed entomopathogenic properties in *G. mellonella*, while in Sweden, it behaved as a saprophyte and competed with other nematodes for insect cadavers (Campos‐Herrera et al., 2012; Torrini et al., 2015). Similarly, *R. regina* collected in Mexico was entomopathogenic against coleopterans of the genera *Phyllophaga*, *Cyclocephala*, and *Paranomala* (Jiménez‐Cortés et al., 2016), but *R. regina* collected from the Netherlands presented phoretic associations with the beetle *Nicrophorus vespilloides* (Wang & Rozen, 2019). The observed variability can be elucidated through the lens of the geographic mosaic (Thompson, 2010), which proposes that interactions diverge according to geographic variation. Each distinct environment imposes unique selective pressures, shaping biotic interactions and giving rise to distinct symbiotic relationships. Nematode traits, such as tolerance to specific bacterial species or the capacity for bacterial retention, are likely to vary across the different geographic mosaic. Similarly, factors such as bacteria colonizing abilities, entomopathogenic potential, and soil bacterial diversity may exhibit fluctuations. These variations can significantly influence the associated microbiota, lifestyle, and entomopathogenic characteristics of rhabditids.

If the environmental conditions are enabling entomopathogeny emergence, and so are the costs and benefits implicated in the association, then a coevolutionary mutualism would gradually evolve. For instance, certain selective pressures may be maintaining *R. regina* symbiosis with *S. marcescens* or *Bacillus thuringiensis*, and therefore its entomopathogenic property. In contrast, weak associations promote bacteria exchange with others present within the habitat (Jiménez‐Cortés et al., 2016; Midha et al., 2017). This holds significance in the life histories of rhabditids, as novel interactions may benefit one trait linked to fitness while simultaneously negatively impacting another, thus generating evolutionary costs (Poulin, 2007; Zera & Harshman, 2001). Considering this, the stability of the associated microbiome under emerging selective pressures could be assessed, such as the colonization of new niches or the acquisition of new bacteria. Additionally, studying the life history of rhabditids whose versatility in lifestyles (e.g., facultative parasitism) is attributed to the association with specific bacteria is essential. Comparing and analyzing nematode fitness components in different environments will shed light on their adaptive process. We suggest conducting experiments using different combinations of nematodes and bacteria to assess the interactants fitness consequences and the entomopathogenic plasticity. Addressing these research lines will enable the identification of factors linked to the success of rhabditids in their transition through various lifestyles, highlighting the importance of preserving generalized microbiotas in these links that traverse the evolutionary continuum.

## 10 CONCLUSIONS

Convergent evolution within the Rhabditida order has driven the emergence of the entomopathogenic trait in the Rhabditidae, Diplogasteridae, Heterorhabditidae, and Steinernematidae families. However, the entomopathogenic trait is only one of several alternative strategies that these lineages can adopt within their life history, depending on the selective pressures. This feature places them within the evolutionary continuum of rhabditids, where the specialization demonstrated by Heterorhabditidae and Steinernematidae represents an alternative evolutionary path to the commensal, generalist, and weak symbioses found among the *Caenorhabditis*, *Oscheius*, *Heterorhabditoides*, *Pristionchus*, and *Rhabditis* lineages. These lineages may represent the incipient and intermediate stages of this evolutionary path. Further studies are needed on these later species to demonstrate the stability of the entomopathogenic nature and to deepen our understanding of the symbioses they form with bacteria. Identifying the effect of these associations on nematodes will support the idea that these organisms may not only behave as genuine entomopathogens but may also respond effectively to the environmental pressures that influence a plastic life history. Although some species don't exhibit the same mutualistic nature of *Steinernema* and *Heterorhabditis* with their symbionts, it does not mean that the entomopathogenic lifestyle is exclusive to a few lineages. Any association between organisms that mutually benefit will give rise to co‐speciation processes or events such as coevolution, where symbionts share adaptive changes and a convergent life history. Studying all these symbiotic relationships provides an opportunity to test theories related to symbiosis (Douglas, 2008), including how costs and benefits within each association vary depending on the environment and symbiont; how specific symbionts are transmitted between generations; how tolerance or evasion of the host's immune response is achieved, and how symbiosis (considering the potential role of fungus and virus) acts on the evolution of this fascinating Phylum: Nematoda.

## AUTHOR CONTRIBUTIONS

**V. J. Trejo‐Meléndez:** Conceptualization (lead); writing – original draft (lead); writing – review and editing (lead). **J. Ibarra‐Rendón:** Conceptualization (equal); writing – review and editing (equal). **J. Contreras‐Garduño:** Conceptualization (equal); funding acquisition (lead); project administration (lead); supervision (lead); writing – original draft (equal); writing – review and editing (equal).

## FUNDING INFORMATION

This study was realized with the support of Programa de Apoyo a Proyectos de Investigación e Innovación Tecnológica, DGAPA, UNAM No. IN225120 provided to JCG. CONAHCyT provided a grant for a scholarship to VTM 850467.

## CONFLICT OF INTEREST STATEMENT

The authors confirm that they have no conflict of interests.

## Supporting information


Appendix S1.

## Data Availability

This review has no data to provide.
