# Supplementary material for: The evolution of entomopathogeny in nematodes
Source: Ecol Evol. 2024 Feb 13;14(2):e10966. doi: 10.1002/ece3.10966 (PMC10862191; doi:10.1002/ece3.10966)
Supplement: Supplementary file 1 — Appendix S1. [file ECE3-14-e10966-s001.docx]

**Box 1. Rhabditids biological attributes that favor the evolution of entompathogeny**

1. Exaptation and the evolutionary continuum

According to Osche (1956) and Sudhaus (2008, 2010), saprophytic nematodes possess adaptations that enable them to tolerate and counteract the conditions of saprophytic environments, including hypoxia, fluctuating osmotic pressures, high temperatures (37-44°C), and the toxic activity of exoenzymes synthesized by bacteria. Osche (1962, 1963) referred to these attributes as preadaptations or exaptations, wherein the selective pressure of one environment eventually favors an adaptation in another environment (Gould & Vrba, 1982). For instance, the transition from a saprophytic to a parasitic host adaptation. Thus, the similarities between the conditions of a saprophytic environment and those within a host have facilitated the expansion of the ecological niche of nematodes (Poulin, 2007; Dieterich & Sommer, 2009; Sommer & Ogawa, 2013).

The concept of the evolutionary continuum refers to how the gradual adaptation to the host environment led to the evolution of distinct parasitic strategies (Fig. 3). Various saprophytic species of Rhabditida serve as transitional links towards parasitism, with phoresis playing a crucial role in exposing nematodes to new selective pressures, resulting in alternative associations with insects (Rüm, 1956; Sudhaus, 1976; Nicholas, 1984; Clark, 1994; Ortega-Estrada et al., 2012). The oscillation of rhabditids along the continuum clearly demonstrates evolutionary convergence within the order (Sudhaus, 2010; Warburton, 2010). Thus, each type of association should be regarded as a "model" that can elucidate the sequences and alternative pathways towards endoparasitism, which has occurred repeatedly throughout the phylogeny of saprophytic nematodes. Furthermore, necromeny promotes adaptations arising from the interaction with the host's immune response. As a result, the necromenic association within the continuum drove evolution towards two distinct paths: parasitism and entomopathogenicity (Dillman et al., 2012; Sommer & Ogawa, 2013; Blanco-Pérez et al., 2017; Brivio & Mastore, 2018). Specifically, the evolutionary trajectory towards entomopathogenicity was shaped by the mutualistic association of nematodes with entomopathogenic bacteria.

**Figure 3**. The evolutionary continuum of nematode-insect associations. The flowchart represents the evolutionary continuum of nematode-insect associations, with each association connected by exaptations and survival attributes of rhabditids. Free-living nematodes can be found in marine, freshwater, and terrestrial habitats, and may transiently associate with arthropods, other invertebrates, or even vertebrates. Phoresis. Based on particular adaptations, nematodes can use insects for transport or protection without obtaining nutrients from them. Necromeny. The nematodes enter the insect and wait for it to die from natural causes, acquiring nutrients from the decomposition of the cadaver. Parasite. The nematodes are adapted to obtain nutrients from the live host without causing lethal damage, although some may eventually cause the host's death. Entomopathogenic. The nematodes form a symbiosis with pathogenic bacteria that facilitates parasitizing and rapid death of the host. Since host death is necessary for transmission, this can be considered a parasitoid strategy, as seen in Mermithids and insect clades such as Hymenoptera and Diptera (Poulin & Randhawa, 2015). The figure Is based on Dillman et al. (2012).

1. Nematode-bacteria symbiosis

Bacteria are known to promote the adaptation of their hosts to their occupied niche, and bacteriophagy in rhabditids has led to intriguing symbiotic relationships (Thrall et al., 2007; Feldhaar, 2011; Duperron et al., 2013; Maher et al., 2017). Beneficial (mutualistic) bacterial symbioses are found within Nematoda, and these associations can be either facultative and temporary or stable long-term bonds. Additionally, these associations can be ectosymbiotic (i.e., bacteria attached to the cuticle) or endosymbiotic (i.e., in the digestive tract). It is now understood that the nematode microbiota contributes to their development, defense, reproduction, nutrient acquisition, and particularly, the establishment and utilization of the environments frequented by nematodes (Poinar & Hansen, 1986; Slatko et al., 2010; Murfin et al., 2012).

**Box 2. Origins, phylogenetic relationships, and richness of EPN**

Poinar (1993) postulated that the ancestors of Steinernematidae and Heterorhabditidae separately engaged in mutualistic associations with Gram-negative bacterial lineages that gave rise to *Xenorhabdus* and *Photorhabdus*, during the mid-Paleozoic era (375 million years ago). Based on similarities in male caudal features and buccal capsule, Poinar suggested that *Heterorhabditis* evolved from an ancestor closely related to *Pellioditis* (Dougherty & Nigon, 1949), a lineage of Rhabditida comprising marine and terrestrial bacteriophagous nematodes. Species like *P. pellio* associate in necromeny with earthworms, while species like *P. marina* feed on bacteria and associate with three species of bacteria, namely *Pseudomonas* sp., *Micrococcus* sp., and *Flavobacterium marinum*, presumably owing to the nematode's nutritional requirements (Poinar, 1993; Tietjen et al., 1970). The mutualistic benefits generated by bacterial retention facilitated the evolution of free-living bacteriophagous nematodes such as *P. marina* to an entomopathogenic heterorhabditid. *Steinernema*, on the other hand, is believed to have originated from an ancestor related to *Rhabditophanes* Fuchs, 1930, of the Alloionematidae family in Rhabditida (Poinar, 1993). Saprophytes such as *R. aphodii*, *R. cobbi*, and *R. schneideri* associate in phoresy with beetles (Andrássy, 1983). While molecular data supports the close relationship of *Steinernema* with a larger clade comprising plant-parasitic nematodes and nematodes that feed on fungi and bacteria, their origin remains ambiguous (Stock & Goodrich-Blair, 2008). Phylogenetic studies confirm that Steinernematidae and Heterorhabditidae are distant families of polyphyletic origin, and the similarities in their life histories and behavior are the result of convergent evolution (Poinar, 1993; Adams & Nguyen, 2002).

*Photorhabdus* and *Xenorhabdus* belong of the Enterobacteriaceae family, which is within the Gammaproteobacteria order (γ-Protobacteria) (Imhoff, 2005). Phylogenetic analyses indicate that they are sister groups, in contrast to their nematode hosts, which are phylogenetically distant (Tailliez et al., 2010; Stock, 2015; Sajnaga & Kazimierczak, 2020). Recent evidence suggests that the closest living relative of these bacteria is the *Proteus* genus and that their common ancestor lived 200-500 million years ago. However, mutualism with *Steinernema* and *Heterorhabditis* generated different selective pressures that led to the evolution of two bacterial genera with specific associations as a result of stable mutualistic interactions (Boemare, 2002; Chaston et al., 2011; Sajnaga & Kazimierczak, 2020). The genus *Photorhabdus* comprises three recognized species: *P. temperata*, *P. luminescens*, and *P. asymbiotica*. The latter was originally isolated from human wounds but was later found to be associated with a heterorhabditid nematode, of which 18 species have been recorded (Nguyen & Hunt, 2007; Nguyen, 2010; Stock & Goodrich-Blair, 2012). In contrast, there are 22 species of *Xenorhabdus* (Tailliez et al., 2011) and 70 known species of *Steinernema* (Nguyen & Hunt, 2007; Nguyen, 2010; Stock & Goodrich-Blair, 2012).

**BOX 3. The EPNs’ “monogenic” microbiotas**

The literature on entomopathogenic nematodes suggests that their microbiota is monogenic, meaning that nematodes associate with only one specific bacterium (Poinar & Tomas, 1966; Poinar et al., 1977; Dillman et al., 2016). It has been demonstrated that a specific and restrictive relationship exists between *Heterorhabditis* and *Photorhabdus*, where each nematode species associates with a specific bacterial species. Conversely, the symbiosis *Steinernema*-*Xenorhabdus* is less restrictive, allowing different bacterial species to associate with the same nematode species (Akhurst & Boemare, 1990; Adams et al., 2006). However, these studies solely focus on the bacteria of interest and do not account for the possibility of other bacterial lineages, as observed in other bacteriophage nematodes (Dirksen et al., 2020; Backes et al., 2021).

The mutualistic bacteria of EPNs play an essential role in several biological attributes of these nematodes (Poinar & Thomas, 1966; Han & Ehlers, 1998). For instance, in *H. bacteriophora*, axenic dauer larvae (i. e. without their symbiont) are less virulent and do not reach sexual maturity (Han et al., 1991). On the other hand, axenic dauer larvae of *Steinernema* sp. remain virulent, mature, and reproduce, albeit with a reduced yield of progeny in both quantity and quality (Boemare et al., 1996; Ciche et al., 2006). Nevertheless, replacing these bacteria with others maintains the development of nematodes, but the association does not persist and leads to negative effects on their fitness (Akhurst & Boemare, 1990; Ehlers et al., 1990; Han & Ehlers, 1998; Sajnaga & Kazimierczak, 2020).

At present, there is a limited number of studies investigating the microbiota of EPNs. For instance, it has been documented that some species of *Steinernema* associate with bacteria such as *Pseudomonas aureofaciens*, *P. fluorescens*, *Enterobacter agglomerans*, *Serratia proteomaculans*, *Alcaligenes* sp., *Acinetobacter* spp., *Achromobacter*, *Ochrobactrum*, *Deftia*, and others (Lysenko & Weiser, 1974; Boemare et al., 1983; Ogier et al., 2020). Moreover, for species of *Heterorhabditis*, an association with the bacterium *Providencia rettgeri* has also been reported (Jackson et al., 1995). While these studies do not investigate the bacteria's function, they lend support to the notion of a diverse microbiota. Nonetheless, studying nematodes in laboratory conditions prevents us from knowing the natural state of their microbiota and how robust or fragile its structure is.

The investigation of the natural microbiota of EPNs is paramount as it provides insight into the genuine interplay between nematodes and their microbiota. The lack of this knowledge precludes us from understanding how the microbiota responds to changes that occur to the nematode under laboratory conditions and the subsequent implications. For instance, the microbiota of wild-type dauer larvae of the entomopathogenic nematode *Rhabditis regina* underwent changes in its composition when maintained in laboratory conditions; however, certain bacterial lineages such as *Serratia marcescens*, *Bacillus thuringensis*, and *Klebsiella* sp. are retained across multiple generations (Jiménez-Cortés et al., 2016). The persistence of other commensal bacteria may be attributed to their fundamental functions, such as offering special protection against pathogens (Saphira, 2017). Studies in *C. elegans* have demonstrated that Gammaproteobacteria such as *Pseudomonas* or *Enterobacter* safeguard the nematode against fungal and bacterial infections (Dirksen et al., 2016; Félix & Duveau, 2012). Additionally, the microbiota exhibits metabolic capabilities that provide the host with essential nutrients, which may also explain the retention of particular bacterial species. Endowing specific metabolic capabilities is a crucial property of the microbiota that has profound implications for the ecology and evolution of the host. For instance, in *C. elegans*, the metabolic attributes of its microbiota align with its saprophytic lifestyle, with strains of *Paenibacillus* and *Bacillus* capable of breaking down polysaccharides such as cellulose, mannan-oligosaccharide, and rhamnogalacturonan (Zimmermann et al., 2020). This metabolic synergy is crucial for the nematode as it primarily colonizes decomposing fruit. From an ecological and evolutionary standpoint, considering how alterations in the microbiota composition could either facilitate or constrain niche colonization, thereby influencing the nematode's adaptation, is of top importance (Henry et al., 2013; Maher et al., 2017).

Undoubtedly, comprehending the diversity and dynamics of the microbiota in EPNs and other rhabditids would aid in the ecological and evolutionary understanding of this group. Generate a mutualism with new bacterial partners may confer novel adaptive strategies, yet it may also entail trade-offs related to adaptation (Maher et al., 2017). Investigating this phenomenon would elucidate the evolutionary trajectory leading to similar associations as those found in *Heterorhabditis*-*Photorhabdus* and *Steinernema*-*Xenorhabdus*.

**BOX 4. The requirements of a bacterial symbiont**

To facilitate coevolution between nematodes and bacteria, bacteria must develop specialized adaptations that prevent their destruction by the nematode's digestive enzymes and promote their establishment within the nematode host (Poinar, unpublished data). Previous research on EPN has suggested that the retention of their natural bacterial symbionts is due to specific molecular adhesions between the glycocalyx and bacterial cilia and lectins expressed on the nematode's surface (Brehelin et al., 1993; Moureaux et al., 1995). In *Steinernema carpocapsae* and its bacterial symbiont *Xenorhabdus nematophila*, it is known that the nematode’s bacterial vesicle provides essential nutrients in the form of amino acids (histidine, serin, leucin, isoleucine and valine) and vitamins (nicotinate and pantothenate). The bacteria cannot synthetize these nutrients and uses them during the dauer stage (Goodrich-Blair, 2007). Molecular studies have identified ten genes in *X. nematophila* which enable its colonization in *S. carpocapsae* by coding for structural and regulatory proteins of metabolism, with three genes (*nilA*, *nilB*, and *nilC*) of particular interest. These genes encode cell wall proteins that allow for interaction with the epithelium of the nematode's digestive tube and facilitate nutrient acquisition (Ciche et al., 2006). It is interesting that homologous genes to *nilB* have also been found in other γ-proteobacteria, such as a strain of *Serratia* associated with *C. briggsae*, suggesting that convergent evolution could potentially link other bacterial lineages with nematodes (Heungens et al., 2002; Cowles & Goodrich-Blair, 2008; Abebe-Akele et al., 2015). Comparative genome analysis between *P. luminescens*, *P. asymbiotica*, *X. nematophila*, and *X. bovienii* has revealed that similarities in gene products that benefit the nematode are due to convergence, such as the synthesis of antimicrobial components that limit the growth of competing microbes (Duchaud et al., 2003; Wilkinson et al., 2009; Chaston et al., 2011). In contrast, genes involved in the entomopathogenic character, such as those that encode for insecticidal toxins, appear to be conserved among the four bacterial species (Chaston et al., 2011).

On the other hand, the interaction between nematodes and their symbionts is a result of their immune response. Although this phenomenon has not been extensively studied in entomopathogenic nematodes, it has been investigated in other rhabditids. For instance, in *C. elegans*, the insulin signaling pathway has been shown to define the assembly of its microbiota by activating particular immune responses (Dirksen et al., 2016; Zhang et al., 2021). While the establishment of symbionts suggests immune tolerance by nematodes, a deeper understanding of the mechanisms involved in the molecular dialogue between host and symbiont is necessary (Schneider & Ayres, 2008). Furthermore, transcriptomic and proteomic studies of the symbionts of EPNs revealed various antibiotics, adhesins, hemolysins, proteases, and lipases that are essential for infecting and transforming insect cadavers (Bode, 2009). Genomic studies of the *Serratia* strain found in *C. briggsae* also showed several genes that encode for entomopathogenic proteins (Abebe-Akele et al., 2015). Interestingly, the absence of Tc complexes (toxin complexes) and Mcf genes (makes caterpillars floppy, which code for lethal toxins for the insect) in *Serratia* suggests the use of different mechanisms or strategies to kill insects (Abebe-Akele et al., 2015).

It is worth noting that *Xenorhabdus* and *Photorhabdus* share similarities in their genetic products, such as the O-antigen, the syrP protein, various non-ribosomal synthetases, bacteriocins, ciliary biogenesis and coupling proteins, secondary metabolites, toxin secretion systems, and multiple drug resistance. There are regions of the genetic code of *Serratia*, *Xenorhabdus*, and *Photorhabdus* whose function is unknown, but it is suggested that they contribute to their association with nematodes. However, further research is needed to better understand these genes (Chaston et al., 2011).

Studies in the "omics" era, such as genomics, transcriptomics, and proteomics, hold the promise of uncovering the underlying mechanisms involved in scenarios of response, resistance, or tolerance to associated bacteria. This, in turn, would enable us to better understand the molecular dialogue between host and symbiont. Additionally, these studies could answer several other pertinent questions regarding aspects of the mutualistic relationship, including: how do nematodes respond to the physical presence of bacteria on their cuticle? How do they differentiate between various types of bacteria, and select beneficial associations over pathogenic ones? And, what is the dynamics of the symbiont community?
